# Supplementary material for: Comparative Mitogenomics of Flesh Flies: Implications for Phylogeny
Source: Insects. 2022 Aug 10;13(8):718. doi: 10.3390/insects13080718 (PMC9408989; doi:10.3390/insects13080718)
Supplement: Supplementary file 1 [file insects-13-00718-s001.zip › insects-1826472-supplementary.pdf]

Supplementary Materials

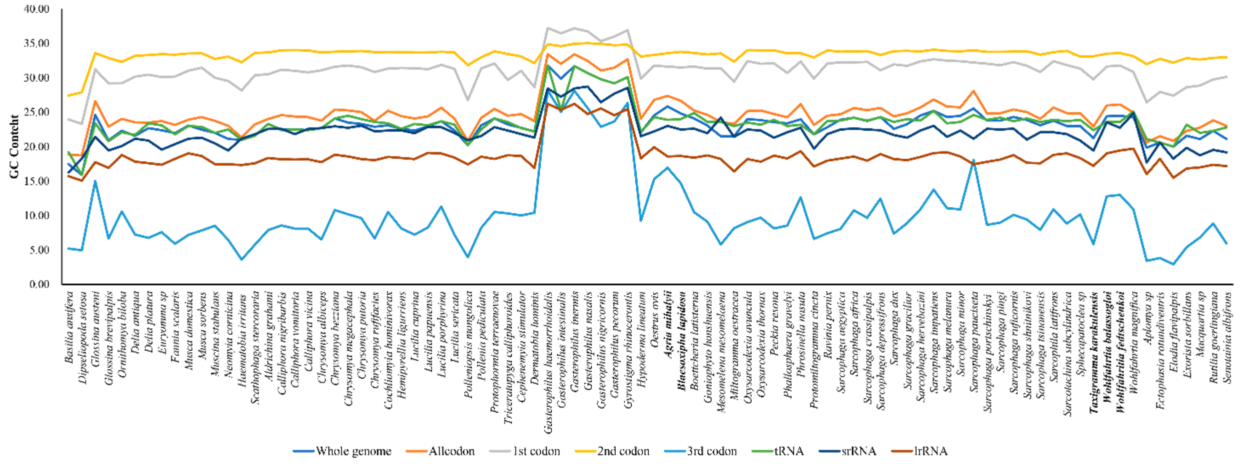

Figure S1. GC content of 13 mitochondrial protein-coding genes in Calyptratae. (GC: Guanine and Cytosine). The sequences for species with names in bold were generated in this study.

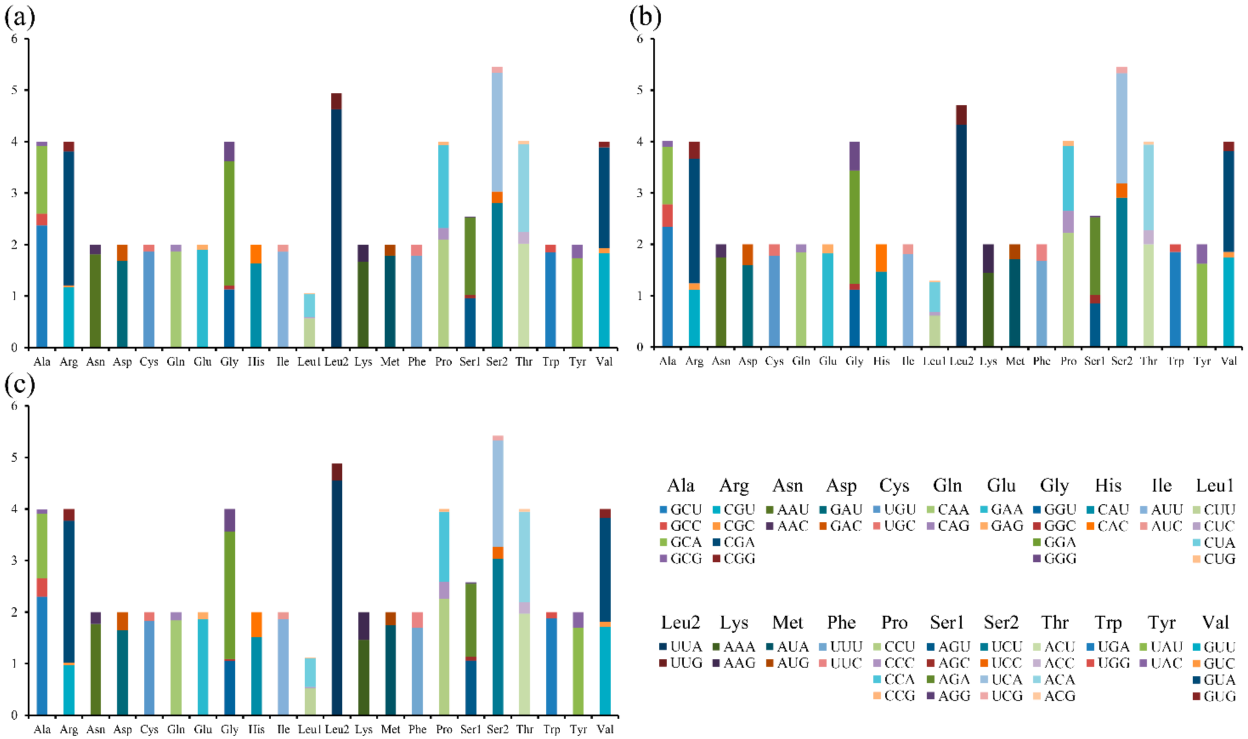

Figure S2. Codon usage analysis of the mitochondrial genomes from three subfamilies (a: Miltogrammatinae, b: Paramacronychiinae and c: Sarcophaginae).

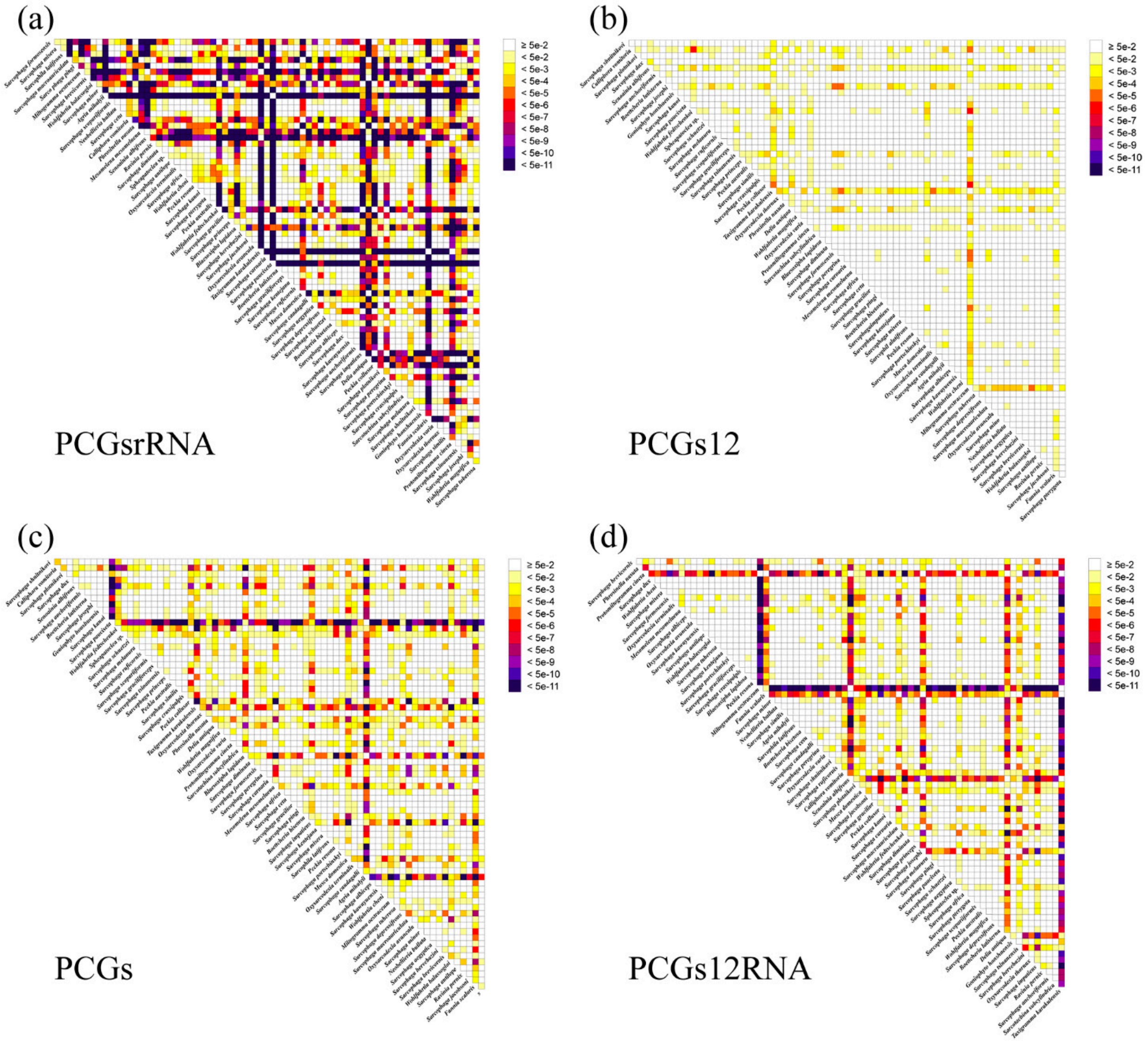

Figure S3. Heatmaps showing pairwise heterogeneous sequence divergence of matrices used for phylogenetic analyses. Abbreviations: PCGsrRNA (a), all 13 protein-coding genes and two rRNA; PCGs12 (b), 1st- and 2nd- codon positions of the 13 protein-coding genes; PCGs (c), all 13 protein-coding genes; PCGs12rRNA (d), 1st- and 2nd- codon positions of the 13 protein-coding genes and two rRNA.

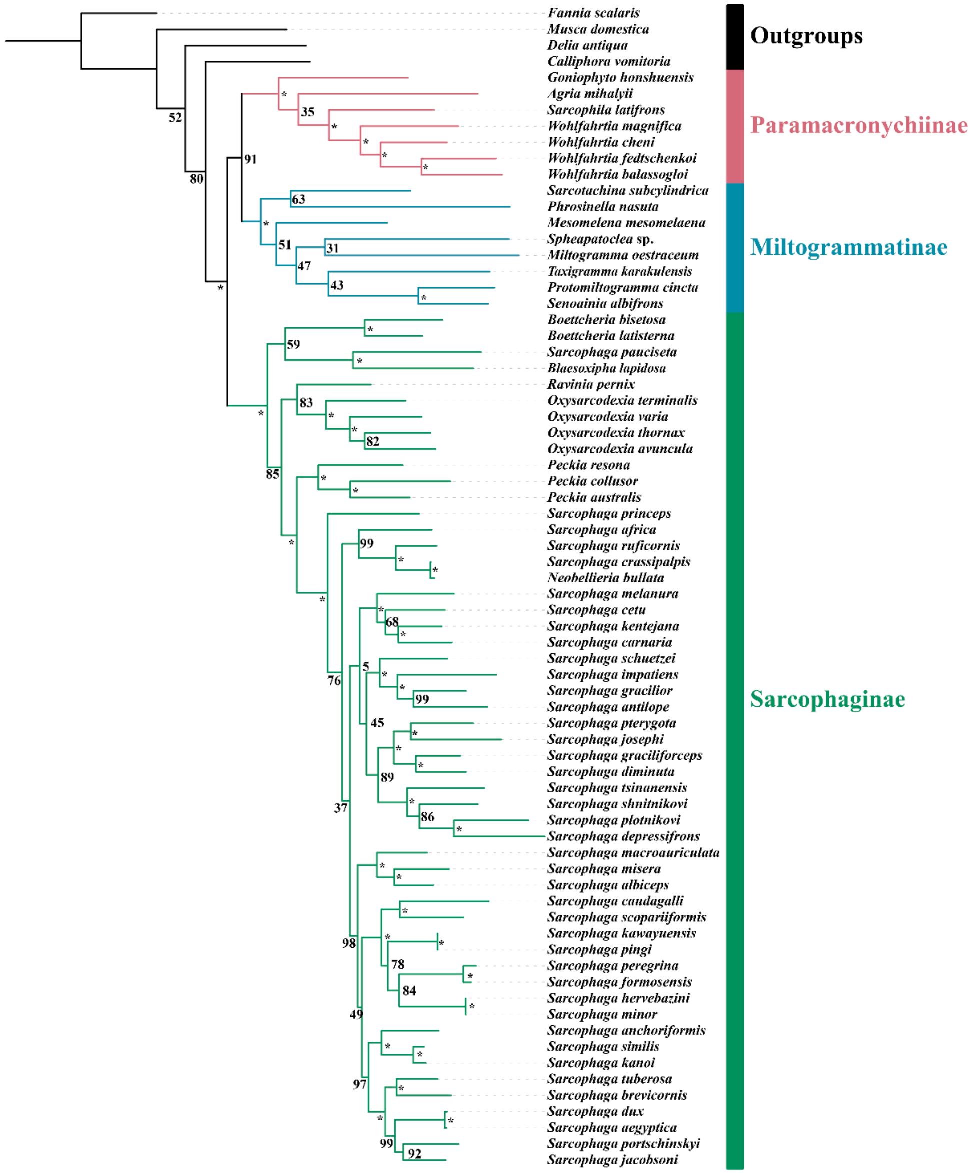

Figure S4. Maximum likelihood phylogenetic tree for Sarcophagidae based on concatenation of 13 mitochondrial protein-coding genes and two rRNA genes. Each node is provided with the support values of Maximum Likelihood bootstrap values (BS) for tree inference using matrix PCGsrRNA. Asterisks (*) represent full support.

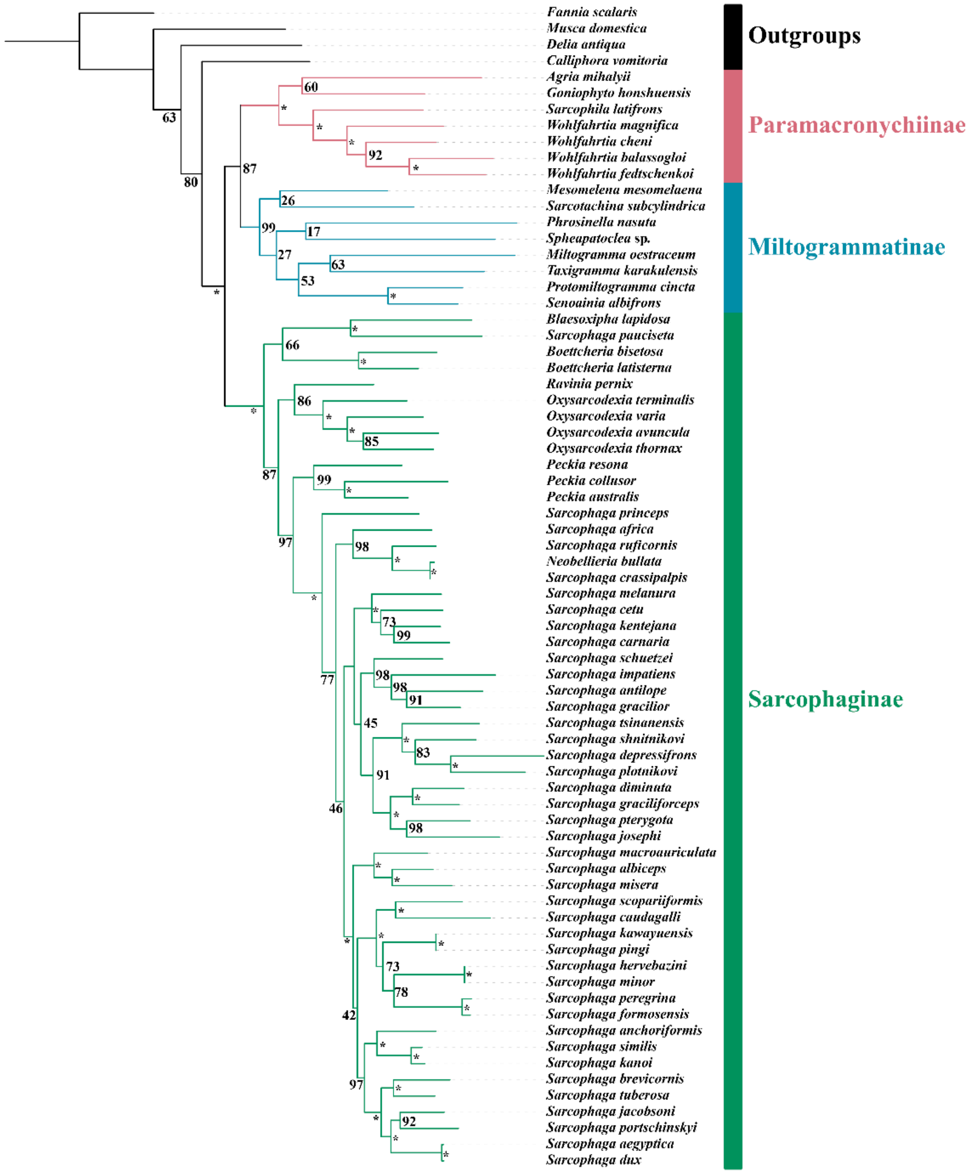

Figure S5. Maximum likelihood phylogenetic tree for Sarcophagidae based on concatenation of 13 mitochondrial protein-coding genes. Each node is provided with the support values of Maximum Likelihood bootstrap values (BS) for tree inference using matrix PCGs. Asterisks (*) represent full support.

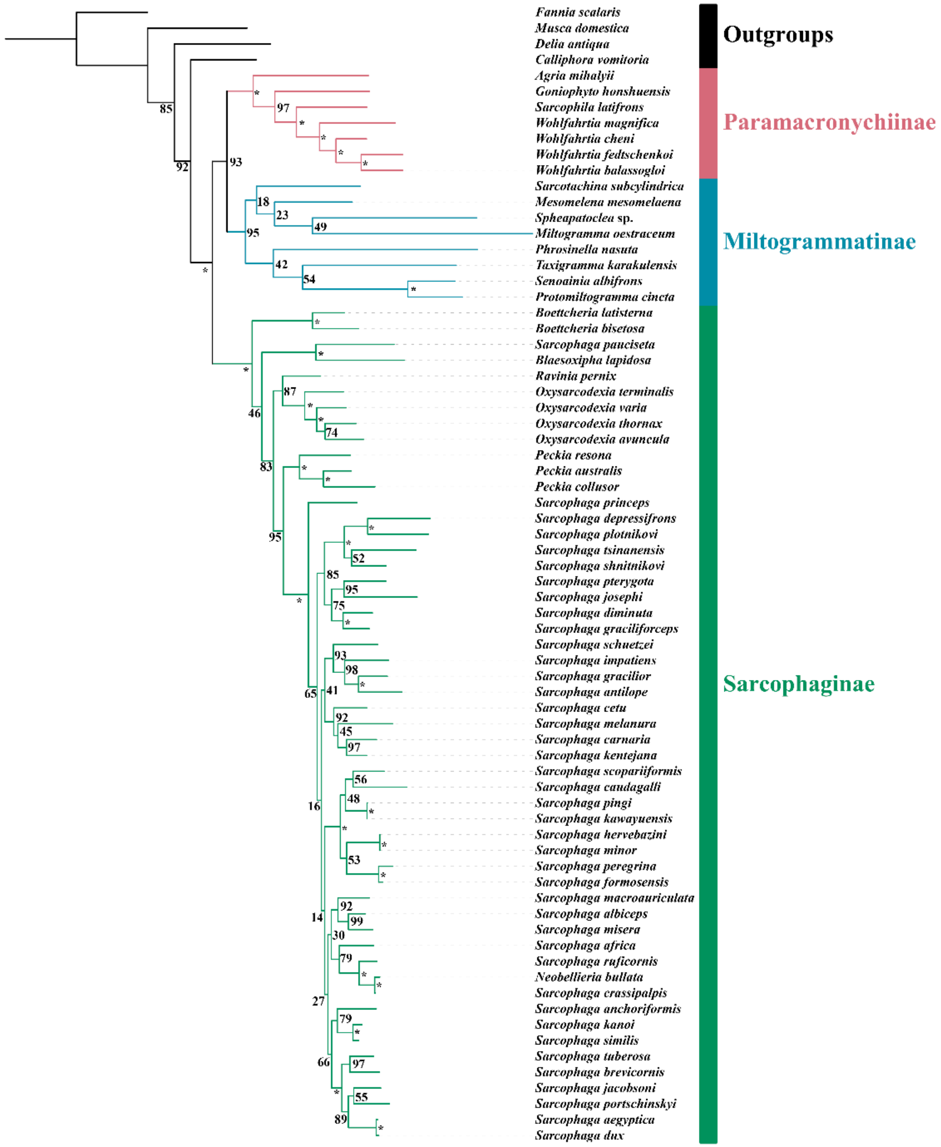

Figure S6. Maximum likelihood phylogenetic tree for Sarcophagidae based on concatenation of 13 mitochondrial protein-coding genes excluding 3rd-codon positions and two rRNA genes. Each node is provided with the support values of Maximum Likelihood bootstrap values (BS) for tree inference using matrix PCGs12rRNA. Asterisks (*) represent full support.

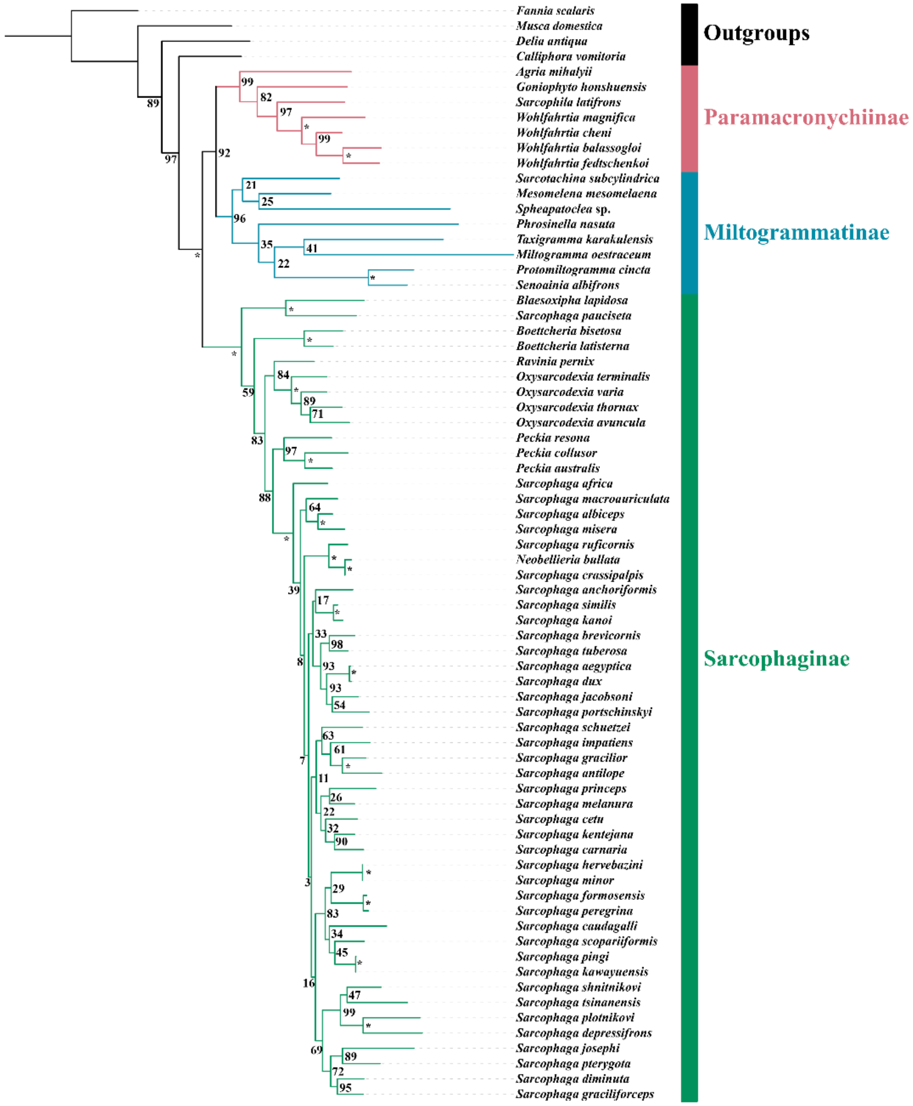

Figure S7. Maximum likelihood phylogenetic tree for Sarcophagidae based on concatenation of 13 mitochondrial protein-coding genes excluding 3rd-codon positions. Each node is provided with the support values of Maximum Likelihood bootstrap values (BS) for tree inference using matrix PCGs12. Asterisks (*) represent full support.

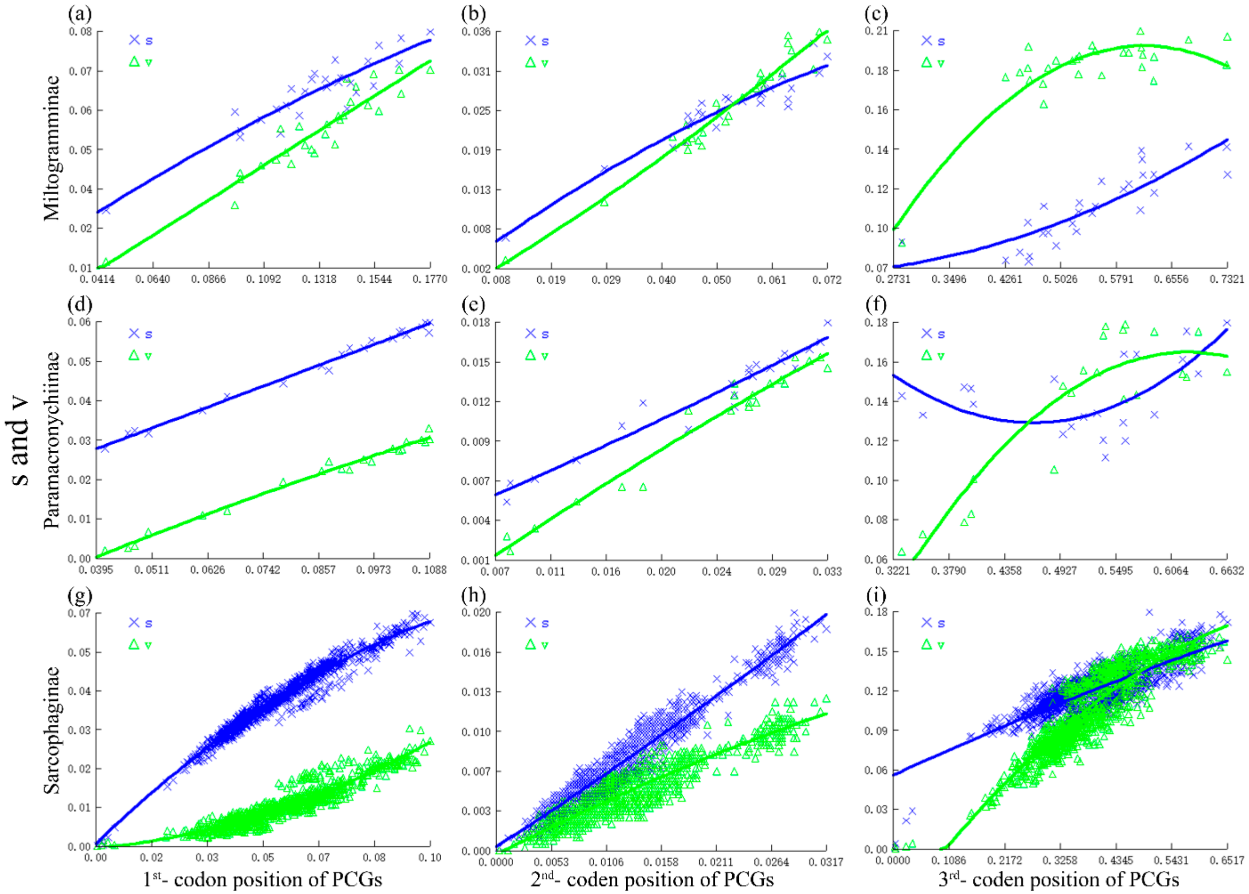

Figure S8. Nucleotide substitution saturation plots of all the 13 PCGs of three subfamilies based on 1st-, 2nd-, and 3rd- codon positions (a, b, c: Miltogrammatinae; d, e, f: Paramacronychiinae; g, h, i: Sarcophaginae). Transition and transversion are represented by blue and green plots, respectively.

Table S1. Collection information of specimens of flesh flies used for DNA extraction in this study.

**Species**	**Collection Location**	**Collection Time**
Agria mihalyii	Songshan, Beijing, China	5-Jun-16
Blaesoxipha lapidosa	Kalamaili Nature Reserve, Xinjiang, China	1-Jul-15
Taxigramma karakulensis	Kalamaili Nature Reserve, Xinjiang, China	29-Apr-16
Wohlfahrtia balassogloi	Kalamaili Nature Reserve, Xinjiang, China	23-Apr-15
Wohlfahrtia fedtschenkoi	Kalamaili Nature Reserve, Xinjiang, China	16-May-15

Table S2. The sequence characteristics of mitochondrial genome of flesh flies.

	Agria_mihalyii				*Blaesoxipha_campestris*				*Taxigramma_karakulensis*				*Wohlfahrtia_balassogloi*				*Wohlfahrtia_fedtschenkoi*			
**Gene**	**Strand**	**Location^{a}**	**Length(bp)**	**Codon Start/Anti Stop**	**Strand**	**Location^{a}**	**Length(bp)**	**Codon Start/Anti Stop**	**Strand**	**Location^{a}**	**Length(bp)**	**Codon Start/Anti Stop**	**Strand**	**Location^{a}**	**Length(bp)**	**Codon Start/Anti Stop**	**Strand**	**Location^{a}**	**Length(bp)**	**Codon Start/Anti Stop**
tRNA^{Ile}	+	1-66	66	AAT	+	1-66	66	AGT	+	1-65	66	AAT	+	1-66	66	AAT		1-66	66	AAT
tRNA^{Gln}	-	64-132	69	TAT	-	64-132	69	TAT	-	63-131	69	TAT	-	64-132	69	TAT	-	64-132	69	TAT
tRNA^{Met}	+	132-200	69	AAA	+	135-203	69	AAA	+	131-199	69	AAA	+	135-203	69	AAA	+	140-208	69	AAA
ND2	+	201-1217	1017	ATT/TAA	+	204-1220	1017	ATT/TAA	+	200-1216	1017	ATT/TAA	+	204-1220	1017	ATT/TAA	+	209-1225	1017	ATT/TAA
tRNA^{Trp}	+	1216-1283	68	AAG	+	1219-1286	68	AAG	+	1215-1281	67	AAG	+	1219-1286	68	AAG	+	1224-1291	68	AAG
tRNA^{Cys}	-	1276-1338	63	GGC	-	1279-1342	64	AGC	-	1274-1335	62	GGC	-	1279-1341	63	GGC	-	1284-1348	65	GGC
tRNA^{Tyr}	-	1352-1417	66	GAT	-	1351-1416	66	GAT	-	1349-1413	65	GAT	-	1350-1415	66	GAT	-	1357-1422	66	GAT
COI	+	1416-2949	1534	TCG/T	+	1415-2948	1534	TCG/T	+	1412-2945	1534	TCG/T	+	1414-2947	1534	TCG/T	+	1421-2954	1534	TCG/T
tRNA^{Leu}	+	2950-3015	66	TCT	+	2949-3014	66	TCT	+	2946-3011	66	TCT	+	2948-3013	66	TCT	+	2955-3020	66	TCT
COII	+	3023-3710	688	ATG/CTT	+	3019-3703	685	ATG/T	+	3015-3699	685	ATG/T	+	3019-3706	688	ATG/T	+	3026-3713	688	ATG/T
tRNA^{Lys}	+	3711-3780	70	CAT	+	3707-3777	71	CAT	+	3703-3773	71	CAT	+	3707-3776	70	CAT	+	3714-3784	71	CAT
tRNA^{Asp}	+	3781-3847	67	AAA	+	3777-3843	67	AAA	+	3783-3847	65	AAA	+	3777-3844	68	AAA	+	3785-3851	67	AAA
ATP8	+	3848-4012	165	ATT/TAA	+	3844-4008	165	ATT/TAA	+	3848-4012	165	ATT/TAA	+	3845-4006	162	ATC/TAA	+	3851-4012	162	ATC/TAA
ATP6	+	4006-4683	678	ATG/TAA	+	4002-4679	678	ATG/TAA	+	4006-4677	672	ATG/TAA	+	4000-4677	678	ATG/TAA	+	4006-4683	678	ATG/TAA
COIII	+	4683-5471	789	ATG/TAA	+	4679-5467	789	ATG/TAA	+	4677-5465	789	ATG/TAA	+	4677-5465	789	ATG/TAA	+	4683-5471	789	ATG/TAA
tRNA^{Gly}	+	5478-5542	65	ATC	+	5474-5538	65	ATC	+	5468-5532	65	ATT	+	5468-5532	65	ATC	+	5478-5542	65	ATC
ND3	+	5543-5896	354	ATT/TAA	+	5536-5892	357	ATA/TAA	+	5533-5886	354	ATT/TAA	+	5533-5886	354	ATT/TAA	+	5543-5896	354	ATT/TAA
tRNA^{Ala}	+	5921-5988	65	AGG	+	5895-5959	65	AGG	+	5887-5949	63	AGG	+	5889-5952	64	AGG	+	5899-5963	65	AGG
tRNA^{Arg}	+	5989-6051	63	GAA	+	5959-6022	64	GAA	+	5950-6013	64	GAA	+	5953-6015	63	GAA	+	5964-6026	63	AAT
tRNA^{Asn}	+	6052-6116	65	TTA	+	6024-6088	65	TTA	+	6015-6078	64	TTA	+	6017-6081	65	TTA	+	6026-6090	65	TTA

tRNA Ser	+	6117-6184	68	GAA	+	6088-6157	70	AGA	+	6079-6146	68	GAA	+	6082-6149	68	GAA	+	6091-6158	68	GAA
tRNA Glu	+	6185-6251	67	ATT	+	6157-6222	66	ATT	+	6154-6218	65	ATT	+	6151-6217	67	GTT	+	6159-6226	68	ATT
tRNA Phe	-	6270-6335	66	ATT	-	6241-6306	66	ATT	-	6237-6303	67	ATT	-	6236-6300	65	ATT	-	6245-6309	65	ATT
ND5	-	6336-8055	1720	ATT/T	-	6307-8023	1717	ATT/T	-	6304-8023	1720	ATT/T	-	6301-8020	1720	ATC/T	-	6310-8029	1720	ATC/T
tRNA His	-	8071-8134	64	ATT	-	8039-8101	63	TTA	-	8039-8102	64	ATT	-	8036-8101	66	ATT	-	8040-8105	66	AAT
ND4	-	8133-9473	1341	ATG/TAT	-	8104-9442	1339	ATG/T	-	8101-9441	1341	ATG/TAT	-	8102-9440	1339	ATG/T	-	8111-9449	1339	ATG/T
ND4L	-	9467-9763	297	ATG/TAA	-	9436-9732	297	ATG/TAA	-	9435-9731	297	ATG/TAA	-	9434-9730	297	ATG/TAA	-	9443-9739	297	ATG/TAA
tRNA Thr	+	9766-9830	65	GTT	+	9735-9799	65	GTT	+	9734-9798	65	GTT	+	9733-9797	65	GTT	+	9742-9806	65	GTT
tRNA Pro	-	9831-9896	66	CAA	-	9800-9864	65	CAA	-	9799-9863	65	CAA	-	9798-9863	66	CAA	-	9807-9872	66	CAA
ND6	+	9899-10423	525	ATT/TAA	+	9867-10391	525	ATT/TAA	+	9866-10390	525	ATT/TAA	+	9866-10390	525	ATT/TAA	+	9875-10399	525	ATT/TAA
CYTB	+	10423-11559	1137	ATG/TAG	+	10391-11527	1137	ATG/TAG	+	10390-11526	1137	ATG/TAG	+	10390-11526	1137	ATG/TAG	+	10399-11535	1137	ATG/TAG
tRNA Ser	+	11558-11625	68	AGT	+	11526-11593	68	AGT	+	11525-11589	65	AGT	+	11525-11592	68	AGT	+	11534-11601	68	AGT
ND1	-	11642-12580	939	ATA/TAA	-	11610-12548	939	ATA/TAA	-	11611-12531	921	ATA/TAA	-	11609-12547	939	ATA/TAA	-	11618-12556	939	ATA/TAA
tRNA Leu	-	12591-12654	64	ACT	-	12559-12623	65	GCT	-	12545-12609	65	ACT	-	12558-12623	66	ACT	-	12567-12632	66	ACT
lrRNA	-	12651-13979	1329		-	12620-13947	1328		-	12606-13934	1329		-	12621-13944	1324		-	12632-13953	1322	
tRNA Val	-	13976-14047	72	CAA	-	13948-14019	72	CAA	-	13935-14006	72	CAA	-	13945-14016	72	CAA	-	13954-14025	72	CAA
srRNA	-	14048-14830	783		-	14020-14804	785		-	14007-14790	784		-	14017-14800	784		-	14026-14815	790	

[a] Strand indicates the strand by which the genes are encoded; plus (+) and minus (-) symbols represent major and minor strands, respectively.

Table S3. Nucleotide composition of the mitogenomes of flesh flies.

Dataset	Species	A%	T%	AT%	AT Skew	G%	C%	GC%	GC Skew
Whole genome	*Agria mihalyii*	38.51	35.92	74.43	0.03	9.91	15.66	25.57	-0.23
	Blaesoxipha lapidosa	39.06	36.11	75.17	0.04	9.52	15.31	24.83	-0.23
	Taxigramma karakulensis	40.08	39.07	79.15	0.01	8.68	12.17	20.85	-0.17
	Wohlfahrtia balassogloi	38.66	37.36	76.02	0.02	9.16	14.82	23.98	-0.24
	Wohlfahrtia fedtschenkoi	38.74	37.19	75.93	0.02	9.25	14.82	24.07	-0.23
All codon	*Agria mihalyii*	29.84	41.97	71.81	-0.17	13.61	14.58	28.19	-0.03
	Blaesoxipha lapidosa	31.26	42.02	73.28	-0.15	13.22	13.50	26.72	-0.01
	Taxigramma karakulensis	32.62	44.39	77.01	-0.15	11.62	11.36	22.99	0.01
	Wohlfahrtia balassogloi	30.91	42.43	73.34	-0.16	13.02	13.64	26.66	-0.02
	Wohlfahrtia fedtschenkoi	30.80	42.35	73.15	-0.16	13.06	13.79	26.85	-0.03
tRNA	*Agria mihalyii*	38.98	37.13	76.11	0.02	11.47	12.42	23.89	-0.04
	Blaesoxipha lapidosa	38.16	37.88	76.04	0.00	12.08	11.88	23.96	0.01
	Taxigramma karakulensis	38.80	38.80	77.60	0.00	11.03	11.37	22.40	-0.02
	Wohlfahrtia balassogloi	38.98	37.41	76.38	0.02	11.54	12.08	23.62	-0.02
	Wohlfahrtia fedtschenkoi	38.80	37.58	76.38	0.02	11.64	11.98	23.62	-0.01
lrRNA	*Agria mihalyii*	39.95	41.46	81.41	-0.02	12.26	6.32	18.59	0.32
	Blaesoxipha lapidosa	39.53	41.72	81.25	-0.03	12.58	6.17	18.75	0.34
	Taxigramma karakulensis	39.80	42.96	82.77	-0.04	11.21	6.02	17.23	0.30
	Wohlfahrtia balassogloi	39.80	41.16	80.97	-0.02	12.61	6.42	19.03	0.33
	Wohlfahrtia fedtschenkoi	39.49	41.07	80.56	-0.02	12.78	6.66	19.44	0.32
srRNA	*Agria mihalyii*	36.91	39.59	76.50	-0.04	15.58	7.92	23.50	0.33
	Blaesoxipha lapidosa	37.45	39.62	77.07	-0.03	15.54	7.39	22.93	0.36
	Taxigramma karakulensis	41.07	38.90	79.97	0.03	12.63	7.40	20.03	0.26
	Wohlfahrtia balassogloi	37.24	38.65	75.89	-0.02	15.43	8.67	24.11	0.28
	Wohlfahrtia fedtschenkoi	37.59	38.86	76.46	-0.02	15.19	8.35	23.54	0.29
Control region	*Agria mihalyii*	42.33	41.04	83.37	0.02	7.51	9.13	16.63	-0.10
	Blaesoxipha lapidosa	49.07	39.75	88.82	0.10	3.42	7.76	11.18	-0.39
	Taxigramma karakulensis	47.40	42.44	89.85	0.06	3.65	6.50	10.15	-0.28
	Wohlfahrtia balassogloi	44.61	43.60	88.21	0.01	3.86	7.94	11.79	-0.35
	Wohlfahrtia fedtschenkoi	46.12	42.01	88.13	0.05	3.99	7.88	11.87	-0.33
